# Mechanism of Acrylate Emulsion-Modified Cement-Based Materials

**DOI:** 10.3390/molecules29061260

**Published:** 2024-03-12

**Authors:** Fuyun Su, Tingshu He, Zhongmao He, Qihao Yu, Haiyan Wang

**Affiliations:** 1 College of Materials Science and Engineering, Xi’an University of Architecture & Technology, Xi’an 710055, China; 2 College of Science & Technology, Ningbo University, Ningbo 315000, China; 3 State Key Laboratory of Frozen Soil Engineering, Northwest Institute of Eco-Environment and Resources, Lanzhou 730020, China; 4 Gansu Zhongyan Tongchuang Material Technology Co., Ltd., Lanzhou 730020, China

**Keywords:** acrylate lotion, cement based, action mechanism, performance improvement, modification

## Abstract

Polymer-modified cement-based materials have been widely used in building materials. Polymers play a crucial role in improving the performance of cement-based materials. At the same time, different polymers are added according to specific special requirements to meet the needs of the industry. Therefore, this paper reviewed the research on the performance and mechanism of acrylic lotion in modifying cement-based materials. Firstly, the role of acrylate lotion in the improvement of the volume stability, mechanical properties, and durability of cement-based materials was discussed to explore the advantages and disadvantages further, optimize the application of polymer in cement-based materials according to the performance improvement, and amplify the advantages of polymer modification. Secondly, the physicochemical mechanism of acrylate-lotion-modified cement-based materials was discussed, and the products and reactants of acrylate lotion in the reaction process of cement-based materials, as well as the interaction mechanism of acrylic lotion and cement hydrates, were clarified. Cement hydration is a crucial step in exploring the mechanism of polymer-modified cement-based materials. Due to the acrylate lotion filled on the cement surface and the physical and chemical interaction between them, the cement hydration is delayed, resulting in the cement retarding phenomenon. This paper describes its mechanism. Finally, the improvement effect of acrylate lotion on the performance of cement-based materials was reviewed, the research methods of mechanism research on acrylate-lotion-modified cement-based materials were evaluated, and suggestions for future research methods were provided.

## 1. Introduction

Cement is a porous and brittle material that tends to crack under external forces [1,2,3], leading to safety hazards and weakening concrete structures over time. Polymer materials are popular in terms of their short-term performance [4], because they possess excellent properties such as anti-corrosion, waterproofing, seismic resistance, wear resistance, and light weight [5,6]. The interaction between cement and polymer particles redefined the microstructure and interface characteristics of traditional cement-based materials, improving bending resistance and durability at the macro level [4,7,8]. With the gradual maturity of polymer-modified concrete technology, various polymers are now added to meet industry-specific performance requirements. For instance, polymer-modified cement mortar is known for its good crack and acid–alkali corrosion resistance [9,10,11,12].

Concrete polymer composite materials have a long history, dating back to 3000–2000 BC [13,14,15]. While natural and semi-synthetic polymers were used in the early days, synthetic polymers have recently become the preferred choice for modifying cement-based materials due to their excellent water solubility stability and ease of preparation and modification [16,17]. In the 1940s, products modified with polyvinyl chloride were developed to enhance the bonding strength between new and old concrete and manufacture concrete pipes [18]. Today, polymer concrete materials are still a new trend in modified cement-based materials [19,20]. Latex polymers such as acrylic ester (PAE), styrene–butadiene rubber (SBR), and polyvinyl alcohol (PVA) are among the non-toxic and environmentally friendly materials used for polymer modification, with high resistance to chemical corrosion and high bonding strength [4,21,22,23]. While there was a misconception in the past that adding any polymer would affect the performance of concrete, this viewpoint has yet to be confirmed. With the progress of scientific research and an understanding of the mechanism of polymer-modified cement-based materials, the development goal of polymer cement concrete has been repositioned [24,25,26].

This paper mainly studies the modification effect of acrylate lotion on cement-based materials, reveals the mechanism of acrylate lotion modifying cement-based materials, and summarizes the influence of polymers on the hydration process of cement-based materials based on the modification mechanism. The microstructure of materials is inevitably changed by polymer lotion. To better control the internal structure of polyacrylate-lotion-modified cement composites and optimize the cement-based performance, it is necessary to conduct a deeper study on the mechanism of polymer-modified cement-based materials. This not only provides a theoretical basis for the further application of acrylate-lotion-modified cement-based materials, but also brings new ideas for the development of new polymer-lotion-modified cement materials.

## 2. Properties of Cement-Based Materials Modified by Acrylate Lotion

Polymer latex is used in modified concrete in the following two ways: 1. Polymer latex without active groups on the polymer chain. It changes the physical properties of cement by covering the surface of cement hydration products and porous areas of cement. 2. Latex has active groups that improve cement’s physical and chemical properties. It forms a three-dimensional network structure during the hydration process of cement [27]. Acrylate lotion polymer is a suitable choice due to its good flexibility, corrosion resistance, elastic deformation performance, compatibility with cement-based materials, high versatility, and sustainability. It meets the requirements of green environmental awareness, resource conservation, and extended infrastructure service life [28,29,30,31,32]. Therefore, acrylate lotion has become the first choice for modified cement-based materials.

### 2.1. Volume Stability

**Figure 1:**
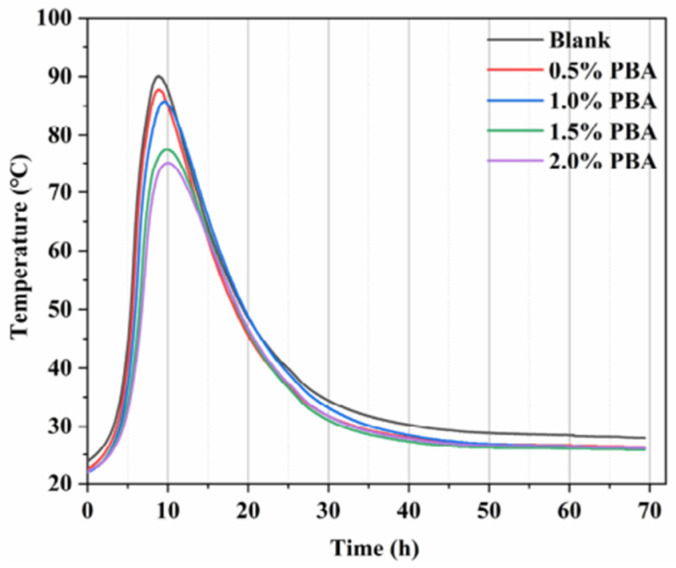
Effect of PBA on rise in cement hydration temperature [37].

In construction engineering, cement hydrates release a lot of heat, which can cause the internal temperature to rise up to 70–80 °C. During the cooling process, the concrete undergoes thermal shrinkage, and the restrained structural concrete generates significant tensile force, ultimately leading to cracking [33,34]. Therefore, controlling the heat of hydration and reducing thermal shrinkage during the cooling process can suppress concrete cracking. Adding polymers reduces the temperature gradient of concrete and the risk of concrete cracking. The heat flow curve of cement hydration is divided into four stages, namely, the initial dissolution stage, induction stage, acceleration stage, and attenuation stage [35,36]. Sifan Li et al. [37] showed that when butyl acrylate (PBA) lotion was added to concrete, the second exothermic peak was mainly concentrated in the cement hydration process, it slowed down due to the increase and decrease rate of PBA addition, and the degree of reduction in the hydration heat peak was positively related to the increase in PBA content (Figure 1). Polymer lotion significantly inhibits the hydration of cement and reduces the heat release of hydration, and the effect of reduction increases with the amount of lotion, thus improving the crack resistance of cement-based materials.

Cement hydration is a complex thermodynamic system where many heterogeneous compounds interact to form cement hydrates. High water-absorbing polymers (SAPs) can be added to improve the internal hardening ability of cement-based materials and reduce self-shrinkage [38]. Lewis et al. [39] studied the thermal analysis of SAP polyacrylic acid acrylamide copolymer suspension cement. The SAP concentration and particle size distribution and their impact on the ordinary Portland cement hydration process were analyzed. The results showed that an increase in SAP concentration was beneficial for forming Ca(OH)_2_ precipitation, and a particle size of 50–75 nm would produce more Ca(OH)_2_. Therefore, the cation exchange and interlocking mechanism in the cementitious solution may influence cement hydration.

Junpeng Mei et al. [40] studied the anti-cracking performance of styrene–acrylic acid lotion (SAE) on cement-based materials. SAE significantly reduces cement-based materials' brittleness coefficient, elastic modulus, elastic strength ratio and drying shrinkage. SAE also reduces the cracking sensitivity of the material. Meanwhile, SAE reduces the number of pores that adversely affect shrinkage, refine the microstructure, and reduce the formation of Ca(OH)_2_ and microcracks in cement-based materials.

There are several reasons for engineering cracks: temperature changes, load, frost heave, foundation deformation, construction process quality, steel corrosion, shrinkage, and material quality. The methods of handling include 1. changing the concrete material itself; 2. using prestressed concrete to reduce load cracks in components; 3. taking repair and reinforcement measures after cracking [41]. The first two methods cannot change rigid materials’ properties, so the crack resistance improvement is limited. The third method is unsuitable for solving the interface problem between new and old materials. Therefore, the treatment of cracks in engineering has not been well addressed yet [42].

With the further hydration of cement, a continuous and closely arranged polymer particle layer is formed on the surface of the cement gel unhydrated cement particle mixture. Cementitious materials and polymer particles will fill larger pores, but the particle size of the polymer is far larger than the pores left by cement hydration. Therefore, the problem between the mortar and the interface is also a complex problem in crack repair. The chemical reactions of polymers such as polyacrylate (PA), acrylate (SAE), and chloroprene rubber (CR) on the surface of the mortar are expected to improve this phenomenon [43,44,45,46]. Binmeng Chen et al. used molecular dynamics to study interface bond structure, kinetics, and mechanical properties between sodium polyacrylate molecules and cement-based materials. The carboxyl groups on polyacrylate provide oxygen sites were shown to receive hydrogen bonds from protonated silicate tetrahedra and adjacent water molecules. Ca^2+^ and Na^+^ act as a bridge between the functional group oxygen atom of hydrated calcium silicate and the silicate chain oxygen. O-Ca-O and O-Na-O salt bridges enhance interface bonding, suppress crack development, and improve the ductility of cement–polymer composites [18,19,20,21,22,23,24,25,26,27,28,29,30,31,32,33,34,35,36,37,38,39,40,41,42,43,44,45,46,47].

### 2.2. Mechanical Properties

The uneven distribution of polymers in cement leads to an incomplete polymer network structure, which significantly reduces the modification effect [48,49,50,51,52]. Additionally, the amount of polymer is also a challenge for polymer-modified cementitious materials. A large amount of polymer improves the water resistance performance of mortar, but it significantly decreases strength [53,54]. There are two ways to add polymers. The first is to increase consistency by adjusting the water–cement ratio, while the second involves the use of admixtures. Adjusting the water–cement ratio is a laboratory procedure, whereas adding additives involves a trial and error process, and its structure can be directly used in practice [55,56].

**Figure 2:**
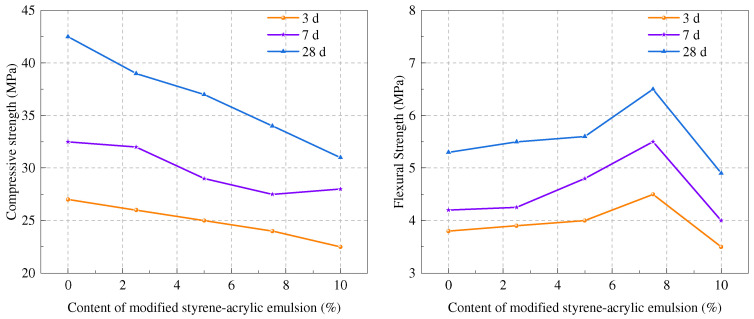
Effect of modified styrene–acrylic lotion on mechanical properties of hardened cement [57].

In Figure 2, Jinxi Dou et al. [57] studied the mechanical properties of cement-based materials through modified styrene–acrylic lotion. When the polymer content was less than 7.5%, the flexural strength significantly increased, but when the content reached 10%, the flexural strength was equivalent to that of the control sample. Zahra Bahranifard et al. [58] investigated the influence of styrene–butyl acrylate (SBA) lotion on concrete. Polymer creates a network which fills the pores of concrete and positively influences its micro-structure. This structure promotes the uniform distribution of materials and improves the compressive strength of cement. Cheng et al. [59] studied the influence of styrene–acrylic acid lotion (SA) on the toughness and wear resistance of cement-based materials. SA can significantly improve the bending and compression properties of materials. At 3%, the maximum bending strength was 10.94 MPa, 49.5% higher than the control sample. The influence of styrene–acrylic acid lotion on the performance of concrete was studied. It was found that the flexural strength of styrene–acrylic acid lotion cement had an increasing trend, and the maximum value at 28 days was 9.7% higher than that of the control sample [60].

### 2.3. Durability

The surface roughness of polymer-modified cement-based materials significantly improves the hydrophobicity of a sample, which helps to improve its durability. Tsigarida et al. studied the effect of material surface roughness on the durability of a sample and found that the surface of the sample had high hydrophobicity after latex treatment, while the durability of the sample was improved [14]. Zhang et al. [61] studied the acid resistance of polymer mortar. Adding styrene–acrylic acid lotion, the corrosion degree of the sample immediately decreased, reducing the loss rate. In a study conducted by Jiang Chao et al. [62], the bonding performance and durability of polyacrylate silica fume mortar were investigated. It was discovered that the polymer effectively enhanced the carbonization resistance of the mortar. Moreover, the improvement effect was more pronounced with an increase in polymer content. Similarly, Zhang Xijun et al. [4] investigated polymer-modified cement mortar and reported that the modified material exhibited excellent waterproof performance, impermeability, water retention, high flexibility, and resistance to acid–alkali corrosion.

The anti-permeability of polymer-modified cement-based materials is attributed to the physical blocking of polymer networks but does not affect the hydrophilicity of the mortar. In recent years, there has been increasing research on the durability of hydrophobic cement-based materials in solutions and freeze–thaw environments, which has become an effective method for enhancing their impermeability [63]. Jin Yang et al. [53] found that the impermeability of concrete mortar modified by styrene–acrylic lotion (SAE) is very strong, but the impermeability is enhanced while the flexural strength is severely lost. Therefore, besides the modification of the polymer itself, secondary modification of the polymer has become a new direction in the research of polymer-modified cementitious materials. In Table 1, the effects of different polymers on the various properties of cement-based materials are compared.

## 3. Mechanism of Acrylate-Lotion-Modified Cement-Based Materials

### 3.1. Mechanism of Acrylate-Lotion-Modified Cement-Based Materials

There are two theories on the mechanism of polymer-modified concrete: 1. There is no interaction between concrete and polymers. During the hydration process of cement, the hydrophilic end of the polymer is close to the water phase, while the hydrophobic end is close to the gas phase. During the drying process, water is evaporated, and hydrophobic polymer particles gather together to form a thin film. 2. Cement hydration products interact with polymers to form a semi-permeable membrane. The Ca(OH)_2_ causes the polymer to form a semi-transparent film. As the amount of Ca(OH)_2_ increases, the amount of film formation decreases, ultimately leading to no film formation. Because Ca^2+^ interacts with carboxyl groups in the polymer, carboxylate ions undergo crosslinking, inhibiting the formation of the film [18,49]. The mechanism process of polymer-modified cement-based materials still focuses on a series of chemical reactions after cement hydration [71,72,73,74,75].

### 3.2. Physical and Chemical Mechanism of Acrylate-Lotion-Modified Cement-Based Materials

There are two views on the mechanism of acrylic acid lotion (PAE) modifying cement substrate: 1. During the hydration process, polyacrylic acid latex covers the surface of cement particles and hydration products or fills cracks in the cement hydration system. These physical effects can improve the porosity of cement. 2. PAE also has chemical reactions and physical behavior. They improve the performance of cement-based materials by connecting chemical bonds through chelation [76,77].

#### 3.2.1. Physical Mechanism of Acrylate-Lotion-Modified Cement-Based Materials

Polymer particles fill the pores of cement-based materials. As the hydration reaction proceeds, these particles accumulate between the pores and the interface transition zones, adsorbing and polymerizing in situ into thin, flexible films. This process forms a network structure that enhances the density and impermeability of cement-based materials, as observed in previous studies [7,78]. Additionally, the interface structures between organic and inorganic materials in polymer-modified cement-based materials create interactions between atoms and molecules in the polymers and the hydration products of cement-based materials through hydrogen bonds, van der Waals forces, and other mechanisms [20].

**Figure 3:**
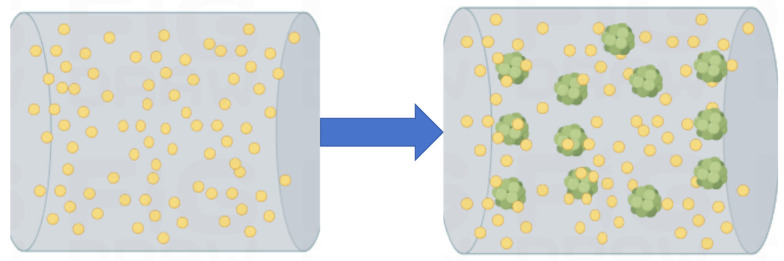
Physical modification simulation of polymer lotion in cement-based materials.

The modification of cement-based materials by polymer lotion occurs locally. Even if more polymer is added, the overall polymer film or network structure will not be formed, but the performance of cement-based materials can be modified at these local locations. This phenomenon is called polymer modification localization [49,79], as shown in Figure 3. Initially, polymer particles, cement particles, water, and sand are mixed together without any physical or chemical reactions, but they are evenly distributed. As the cement hydration reaction progresses, some of the polymers start participating in chemical reactions, while others begin to fill up pores or adsorb onto the surface of cement-based materials, due to adsorption and various ion bonds, which is a physical reaction [80].

#### 3.2.2. Chemical Mechanism of Acrylate-Lotion-Modified Cement-Based Materials

In the second stage of polymer-modified cement-based materials, as the hydration reaction between cement and water progresses, the cement hydration products and Ca^2+^ are released into the pore solution. Some polymers adhere to the surface of cement particles and hydration products, while the other portion combines with Ca^2+^ to form a flocculent structure.

**Figure 4:**
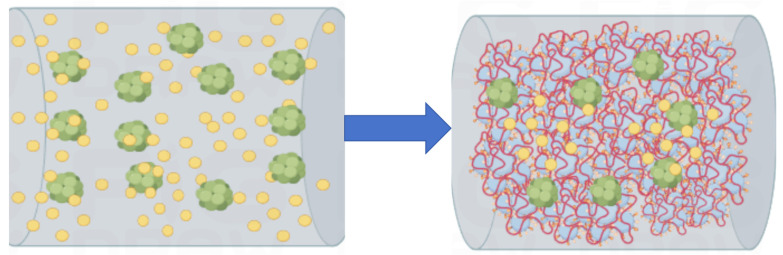
Chemical modification simulation of polymer lotion in cement-based materials.

**Figure 5:**
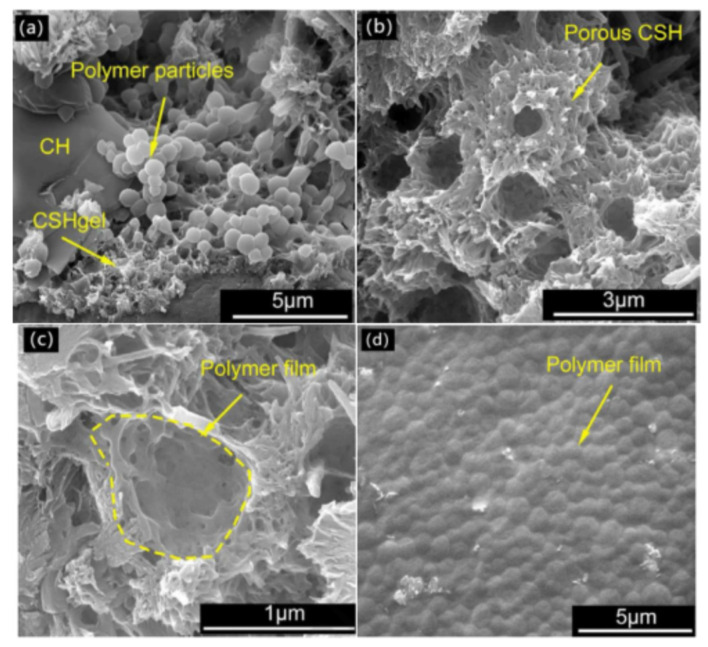
Second electronic image of ethylene vinyl acetate modified cement. (**a**)The absorption of polymer particles on the surface of cement particles; (**b**) porous C-S-H caused by polymer particles; (**c**) the local pore morphology of porous C-S-H; (**d**) formation of a polymer film on the surface of glass [78].

The polymer particles that cover the cement surface will slow down the cement’s hydration process, and eventually, the cement hydrates will break through this barrier. As hydration progresses, the water phase in the pore solution is consumed, and cement hydrates continue to grow or embed into polymer flocs. The accumulated polymer particles will partially accumulate and coagulate into polymer network spatial structures or polymer membranes, as shown in Figure 4 and Figure 5 [81,82].

As early as 1987, the three-step model proposed by Ohama [83] was the most popular in the principle of PMCs (polymer-modified cement-based materials). During the cement hydration process, the polymer forms a continuous and dense thin film on the surface of the cement particles. The adsorbed polymer particles changes the interface structure between the liquid and these particles, thereby affecting the performance of the interface. The interface performance will significantly affect the rheological properties of the cement and further determine the pumpability, self-compaction, and self-leveling of the cement, similar to the cement mixed with water-reducing agents [84,85]. Guo Yanfei et al. [86] studied the influence of polyacrylic acid lotion (PAE) on the fluidity of cement paste and found that when the content of PAE is less than 5.0%, the fluidity is reduced compared to when the content of PAE is more than 5.0%. The addition of PAE combines carboxyl groups with Ca^2+^ in the solution and Ca^2+^ on the surface of cement particles, while polymer PAE adsorbs on the surface of cement particles, both of which may affect the dispersion of the polymer and the rheological properties of PMCs [87,88,89].

The Konietzko model divides the mechanism of polymer-modified cement-based materials into four stages: the uniform dispersion of polymer particles, the accumulation of polymer particles, the aggregation of polymer particles into films, and the formation of spatial network structures by polymers in cement-based materials. The commonality between the Ohma model and the Konietzko model is that the formation of thin films causes the mechanism of polymer modification. But the Ohama model suggests that polymers form a spatial network structure in cement-based materials, and the hardened cement is encapsulated. The Konietzko model suggests that the products of polymer and cement, after hardening, penetrate each other to form a spatial network structure [90,91]. Therefore, both theoretical models are beneficial in explaining the mechanism of polymer-modified cement-based materials.

As further research reveals that the amount of polymer varies, the two theoretical models have different interpretations. When the polymer content is small, it cannot completely wrap the cement-based material, the polymer film forms a three-dimensional network structure with the cement hydration products. When the polymer content is high, the polymer forms a unique network structure to encapsulate cement-based materials. When the amount of polymer decreases, the polymer cannot form a continuous film but is dispersed in cement-based materials [92]. However, the current models established by researchers assume that polymer particles are uniformly dispersed in cement to form a polymer spatial network. The network structure ensures that polymer-modified cement-based materials have good toughness and corrosion resistance. After the polymer and cement paste are mixed, various ions generated by cement hydration impact the polymer’s stability, which may lead to the polymer particles being unable to form a continuous network structure [82,93,94]. Therefore, these models only apply to some of the explanations of the mechanism of polymer lotion-modified cement-based materials.

**Figure 6:**
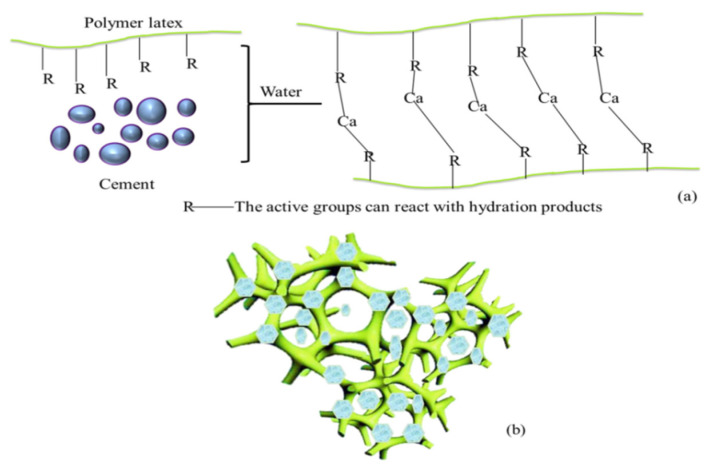
Chemical model of polymer-latex-modified cement. (**a**) The chemical reaction between polymer latex and hydration products. (**b**) A three-dimensional network structure obtained through chemical reactions in polymer-latex-modified cement systems [95].

Wang Ming et al. [76] found three stages of chemical reactions in studying the mechanism of the PAE modification of cement-based materials. Ca(OH)_2_ produced by the hydration of cement leads to the whole system being alkali-rich and exothermic in the first stage, which is very helpful to the hydrolysis of ester groups in the acrylate chain, causing the carboxyl group to be formed in the second stage. In the last stage, the carboxyl group on the polyacrylate lotion chain reacts with Ca(OH)_2_, resulting in the final cross-linking network structure of the product [95]. Cement-based materials were modified using butyl styrene latex (SBR) and carboxyl butadiene styrene latex (XSBRI). After characterization, it was found that no chemical reaction occurred in the SBR latex-modified cement. However, the carboxyl groups in the XSBRI chain react with Ca^2+^ in Ca(OH)_2_, as shown in Figure 6. Hydration products connect the polymer lotion to obtain a three-dimensional network structure, improving polymer-latex-modified cement’s bending strength.

**Figure 7:**
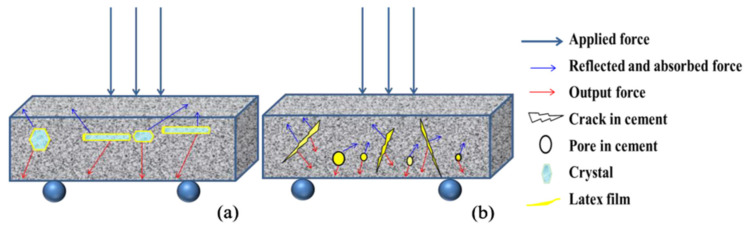
Physical model of polymer-latex-modified cement. (**a**) Polymer latex covers hydrated crystals (**b**) and polymer particles and films are filled in cracks and pores [95].

At the same time, a possible physical modification mechanism model of polymer-latex-modified cement is also established, as shown in Figure 7. Polymer film and particles fill in cracks and pores and reflect some external forces during the fracturing process, improving the flexural strength of cement-based materials. Therefore, the polymer-modified cement-based materials mechanism includes physical and chemical aspects. When no active groups in the polymer can react with hydration products, the modification system only includes the physical modification mechanism. Polymer film covers the surface of crystals and fills the pores, improving the cement’s waterproof performance and bending strength. At the same time, polymer film occupies the position of hydration products, leading to a decrease in compressive strength. However, when the polymer chain of the polymer contains active groups, its mechanisms include physical and chemical modification mechanisms. The physical modification mechanism is the same as that of a polymer without active groups. In the chemical modification mechanism, active groups react with hydration products, connecting polymer latex chains to form a three-dimensional network structure, thereby improving the flexural strength of modified cement [75].

### 3.3. Cement Hydration-Retarding Mechanism in Acrylate-Lotion-Modified Cement-Based Materials

A common disadvantage of polymer lotion is that it hinders the hydration of cement, which will slow the development of concrete strength and even lead to the nonhardening of concrete under extreme conditions. Its retarding principle is the physical and chemical interaction between lotion and cement-based materials [96]. The physical action is the electrostatic interaction between charged polymer particles and the surface of cement-based materials, which drives the adsorption of polymer particles on the substrate surface [97,98]. The chemical action is the complexation reaction between Ca^2+^ in pores and carboxyl groups, which prevents cement hydration [99,100].

**Figure 8:**
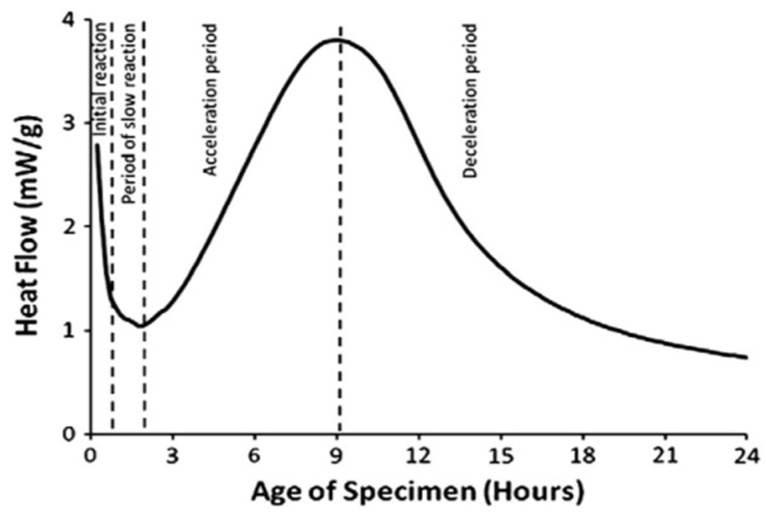
Function of hydration rate over time measured by isothermal calorimetry [101].

**Figure 9:**
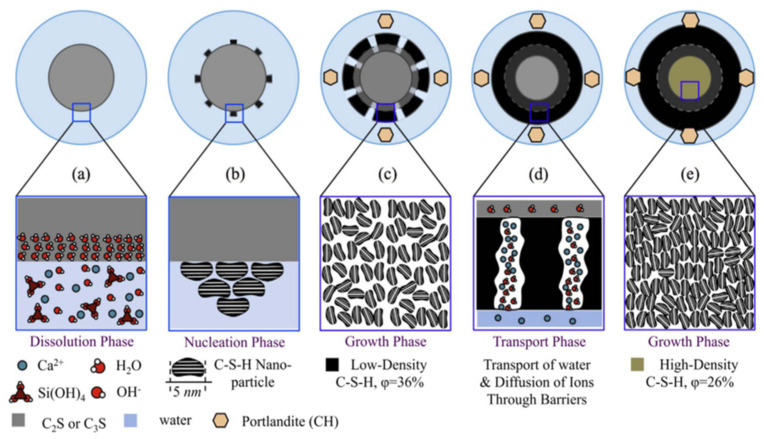
Transport and boundary nucleation growth mechanism of cement hydration [106].

The overall hydration rate of cement can be divided into four stages: (1) initial reaction, (2) slow reaction period, (3) acceleration period, and (4) deceleration period [101,102]. Understanding the hydration mechanism of cement can improve the microstructure of cement hydration and develop new types of cement [103]. The C-S-H formed by cement hydration is the backbone of concrete strength. Due to the complexity of the cement hydration mechanism, controlling the structure and properties of C-S-H remains a challenge. This is also due to the complexity of its structure and chemistry and its interactions during the hydration process of cement [104,105]. In Figure 8 and Figure 9, at each stage of cement hydration, it is assumed that the diffusion mechanism of the spherical C-S-H shell and the growth of C-S-H with the progress of hydration reactions are involved, but more realistic reactions still require future exploration [37,106,107].

**Figure 10:**
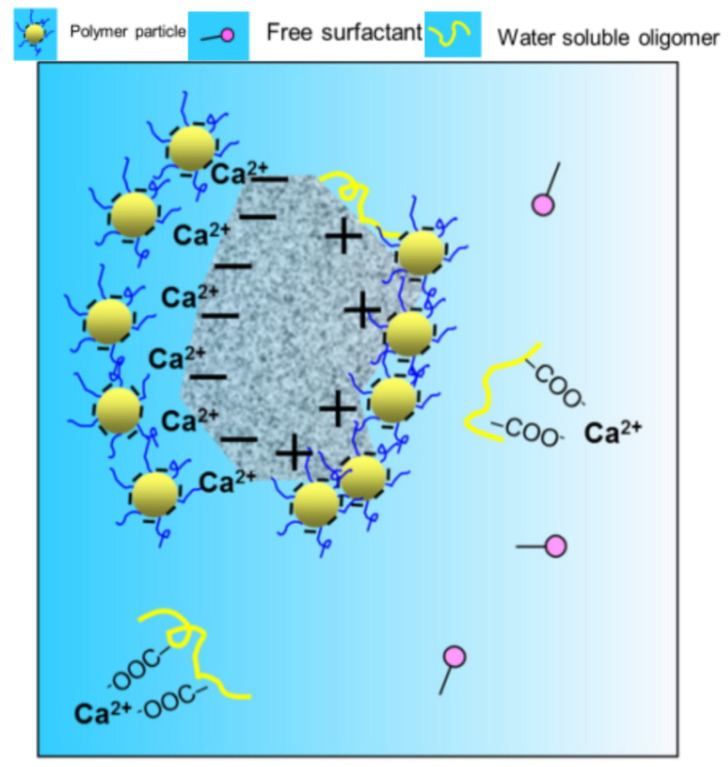
Mechanism of hindrance of polymer latex on cement hydration [95].

Kong Xiangrong et al. [95] took styrene acrylate-based polymer lotion (SA) as an example to reveal the retarding mechanism of cement hydration. Using SA lotion of polymer particles with different surface charges as a model lotion, methacrylic acid (MAA), sodium styrene sulfonate (SSS), and methyl polyethylene glycol methacrylate (MPEGMA) were synthesized as water-soluble monomers (WSMs). They observed whether the surface charge of polymer particles would change due to the use of different water-soluble monomers. Through the characterization of the lotion, it was found that methacrylic acid (MAA) provides carboxyl groups for the surface of polymer particles, and SSS has sulfonic acid groups on the surface of polymer particles. In contrast, MPEGMA has a relatively neutral surface formed by attaching polyepoxyethane (-EO-) chains on the surface of polymer particles. It was found that the “Ca^2+^ capture” in the acrylate polymer should be the reason for the strong retarding effect of the acrylate lotion. Due to the complexation effect, many studies have confirmed the strong interaction between Ca^2+^ and polymer particles with carboxylic acid functional groups. The interaction between Ca^2+^ and polymers containing R-COO− reduces the availability of free Ca^2+^ in pore solutions, which are necessary for forming hydration products such as C-S-H and Ca(OH)_2_. This should be the primary mechanism for extending the guidance period of cement hydration [95,105,108]. During polymer modification, whether the particles in the polymer can adsorb on the surface of cement-based materials is the most crucial step in the retarding behavior of cement hydration. D. S. Hazmmah et al. [109] measured the zeta potential to investigate the influence of anions and cations on the hydration behavior of Portland cement. The results showed that charged particles selectively adsorb on the surface of opposite-charged hydrated cement particles. At the same time, anions in the lotion adsorb positively charged Ca^2+^ in the pores. Research has found an interaction between the polymer and ions (Ca^2+^, SO_4_^2−^, OH^−^) released during the hydration process of cement [110]. It is widely believed that the adsorption between polymer particles and the substrate surface is driven by electrostatic interactions [111], as shown in Figure 10.

In previous studies, styrene acrylate (SA) polymer lotion has had a super retarding effect on cement hydration, which is due to the hydrolysis of butyl acrylate in SA polymer lotion at high temperatures [106,112]. Therefore, modifying the surface of polymer particles to alter the complexation between the polymer and Ca^2+^ is also an essential part of modifying cement-based materials. Zichen Lu et al. [100] modified SA lotion by introducing a PEO hairlike layer and found that polymer particles with a PEO hairlike layer had the smallest retardation on cement hydration. At the same time, it did not affect the hydrolysis of acrylate and the complexation of carboxyl groups generated.

## 4. Conclusions and Outlook

1. Interaction between polymers and cement results in an interpenetrating network structure, which increases the density of cement-based materials and enhances their waterproofing properties, durability, and mechanical strength.

2. The carboxyl group of the acrylate lotion reacts with the cement hydration product, enhancing the cohesion of the cement-based materials and strengthening the structure of the cement-based materials from the inside, thus improving the performance of the cement-based materials.

3. When acrylate lotion improves the performance of cement-based materials, the compressive strength tends to decrease. This may be related to the amount and method of polymer addition, as air may be introduced during the polymer addition process, increasing porosity. During the hydration process of cement, polymers hinder the hydration reaction, resulting in a decrease in strength. However, polymers significantly improve flexural strength, durability, and toughness.

4. Many research experiments have established the physical and chemical mechanisms of polymer-modified cement-based materials. However, there are many types of polymers, and current research has also focused on specific types of polymers, and the established polymer models could be better. At the same time, the experimental conditions, testing methods, and characterization indicators of researchers are different, so the evaluation of results is different.

5. It is necessary to standardize and optimize the design and establish a comprehensive mechanism model, especially for the addition method and dosage of polymers and various controllable strips during the experimental process.

## Figures and Tables

**Table 1:** Effects of different polymers on the properties of cement-based materials.

Polymer Type	Volume Stability	Mechanical Properties
Superabsorbent polymers (SAPs)	SAPs can compensate for the decrease in relative humidity caused by self drying and completely eliminate self shrinkage [64]	SAPs lack a unified and reliable conclusion on the macroscopic mechanical properties of cement-based materials, and their contribution to strength ranges from 20% enhancement to 30% weakening [65]
Styrene–butadiene rubber (SBA)	SBA effectively improves the long-term shrinkage performance of mortar, and the larger the dosage, the smaller the long-term shrinkage deformation [66]	SBA reduces the compressive strength and elastic modulus of concrete [67]
Ethylene–vinyl acetate copolymer (EVA)	Mortar had no volume expansion [68]	EVA reduces the compressive strength of mortar [69]
Polyacrylamide [70]	Added drying shrinkage of cement samples	Anionic PAM significantly reduces the compressive strength and flexural strength of cement

## Abbreviations

Superabsorbent polymers	SAPs
Styrene–butyl acrylate	SBA
Ethylene–vinyl acetate copolymer	EVA
Epoxy resin	EP
Styrene–butadiene rubber	SBR
Polyvinyl alcohol	PVA
Chloroprene rubber	CR
Styrene lotion	SA
Polymer-modified cement-based materials	PMCs
Methacrylic acid	MAA
Sodium styrene sulfonate	SSS
Methyl polyethylene glycol methacrylate	MPEGMA
Water-soluble monomers	WSMs
Carboxyl butadiene styrene latex	XSBRI
